# Plant-Species Diversity Correlates with Genetic Variation of an Oligophagous Seed Predator

**DOI:** 10.1371/journal.pone.0094105

**Published:** 2014-04-11

**Authors:** Liisa Laukkanen, Pia Mutikainen, Anne Muola, Roosa Leimu

**Affiliations:** 1 Department of Biology, Section of Ecology, University of Turku, Turku, Finland; 2 Institute of Integrative Biology, ETH-Zürich, Zürich, Switzerland; 3 Department of Plant Sciences, University of Oxford, Oxford, United Kingdom; University of Calgary, Canada

## Abstract

Several characteristics of habitats of herbivores and their food-plant communities, such as plant-species composition and plant quality, influence population genetics of both herbivores and their host plants. We investigated how different ecological and geographic factors affect genetic variation in and differentiation of 23 populations of the oligophagous seed predator *Lygaeus equestris* (Heteroptera) in southwestern Finland and in eastern Sweden. We tested whether genetic differentiation of the *L. equestris* populations was related to the similarity of vegetation, and whether there was more within-population genetic variation in habitats with a high number of plant species or in those with a large population of the primary food plant, *Vincetoxicum hirundinaria*. We also tested whether genetic differentiation of the populations was related to the geographic distance, and whether location of the populations on islands or on mainland, island size, or population size affected within-population genetic variation. Pairwise F_ST_ ranged from 0 to 0.1 indicating low to moderate genetic differentiation of populations. Differentiation increased with geographic distance between the populations, but was not related to the similarity of vegetation between the habitats. Genetic variation within the *L. equestris* populations did not increase with the population size of the primary food plant. However, the more diverse the plant community the higher was the level of genetic variation within the *L. equestris* population. Furthermore, the level of genetic variation did not vary significantly between island and mainland populations. The effect of the population size on within-population genetic variation was related to island size. Usually small populations are susceptible to loss of genetic variation, but small *L. equestris* populations on large islands seemed to maintain a relatively high level of within-population genetic variation. Our findings suggest that, in addition to geographic and species-specific ecological factors, the plant community affects population genetic structure of oligophagous herbivores.

## Introduction

Herbivores have to adapt to geographically and temporally varying communities of their host plants as well as to genetically determined plant defenses and the nutritive content of their food-plant species. Plant-species composition and plant quality exert selection on herbivores and may affect their population genetics (e.g. [1]). On the other hand, the level of genetic variation in a population may restrict the ability of an herbivore to use certain plant individuals, populations, or species as food (e.g. [2]–[5]). Distinctive selective pressures have promoted some herbivore species to evolve to be dietary generalists and others specialists [6]. Generalists have been observed to harbour higher within-population genetic variation than specialists. The level of genetic differentiation between populations may also differ between specialists and generalists [7]–[9].

The occurrence and nutritive quality of a single food-plant species determine the survival and performance of specialist herbivores (e.g. [10]–[12]). Accordingly, population size, population genetic structure, and secondary chemistry of the primary food-plant species affect the genetic variation and differentiation of populations of specialist herbivores (e.g. [13]). High plant-species diversity may reduce the density of specialist herbivores due to the lower concentration of a certain preferred resource in the environment or due to more abundant and effective enemies [14]–[15]. In contrast to specialists, generalist herbivores with a broad diet may not be strongly affected by a single plant species. Within-population genetic variation and population differentiation of oligophagous herbivores that utilise few food-plant species may be influenced both by the occurrence, abundance, and quality of their primary food-plant species and the diversity of alternative food-plant species in a plant community. We predict that large variety of alternative food-plant species may support stable populations of oligophagous herbivores and thus maintain high level of within-population genetic variation. To our knowledge, there are no studies on the effects of plant-species diversity on the population genetic structure of insect herbivores.

In addition to food-plant quality and diversity, several other, more general, biological and geographic characteristics of the habitat and population may influence the genetic structure of herbivore populations. Small and isolated populations are in general more susceptible to the loss of within-population genetic variation that affects the viability of populations and species [16]. Small and isolated populations may also be more differentiated than large populations or populations with many migrants from other populations [8], [13]. Isolation may be due to geographic distance or the presence of unsuitable habitats for the species to survive or reproduce in [16], [17]. Populations that have persisted over longer times are expected to be genetically more differentiated than younger populations, because random genetic drift, accumulation of mutations, and differential natural selection have had more time to influence their genetic structure [18], [19]. These genetic mechanisms may modify the genetic structure of herbivore populations both independently and in combination with the effects of species interactions. For instance, it is generally expected that genetic differentiation of food-plant populations may lead to differentiation among herbivore populations, but in practice, evidence for this is contradictory (e.g. [20]–[23]).

We investigated the distribution of genetic variation in 23 populations of the oligophagous seed predator *Lygaeus equestris* (Heteroptera) in southwestern Finland and in eastern Sweden. We used amplified fragment length polymorphisms (AFLP) to assess genetic variability. We specifically tested whether genetic variation of *L. equestris* was higher in populations that occurred with large populations of the primary food plant, *Vincetoxicum hirundinaria* [5], [24]. Large *V. hirundinaria* populations may sustain larger and more stable *L. equestris* populations than smaller *V. hirundinaria* populations. Furthermore, while *L. equestris* may use other plant species than *V. hirundinaria* as alternative food [5], [24], we tested whether there was more within-population genetic variation in *L. equestris* populations occurring in habitats with a high total number of vascular plant species. Since *L. equestris* may feed on a large number of alternative food-plant species when the seeds of *V. hirundinaria* are not available ([5], [24], L. Laukkanen, A. Muola and R. Leimu, pers. obs.), we assumed that the total number of plant species correlates with the number of alternative food-plant species in a habitat. A high number of alternative food-plant species may maintain more stable *L. equestris* populations. We also tested whether genetic differentiation between the *L. equestris* populations was related to the similarity of the vegetation in the habitats. Furthermore, we examined how other geographic and ecological factors were related to the genetic structure of the populations. First, we tested whether genetic differentiation between the *L. equestris* populations was related to geographic distance between the populations. We also tested whether island populations had less within-population genetic variation than mainland populations, as migration among the island populations may be limited by water [7], [16]. The size of the island may indirectly affect the viability of the *L. equestris* populations: for instance, large islands may have more diverse habitats for feeding, oviposition, and hibernation compared to smaller islands. Therefore, we tested whether large islands had more genetically variable *L. equestris* populations than small islands. Population size generally influences the level of genetic variation [16], and thus we also tested the impact of population size on within-population genetic variation of *L. equestris*.

## Material and Methods

### Study species

*Lygaeus equestris* L. (Heteroptera: Lygaeidae) is a seed-eating true bug that is specialised to feed on *Vincetoxicum hirundinaria* Med. ( =  *Cynanchum vincetoxicum* (L.) Pers.) (Apocynaceae), a highly poisonous long-lived perennial herb [5], [25]. Although *L. equestris* may occasionally also feed on several other plant species than *V. hirundinaria*, in Scandinavia it is found merely in *V. hirundinaria* populations [24], [26], [27]. *Lygaeus equestris* is locally common in the distribution area of *V. hirundinaria* in Scandinavia, but its population sizes vary considerably among years and populations ([26], [28], L. Laukkanen, unpubl. data). *Lygaeus equestris* is usually univoltine and overwinters as adult. In the study area, the female *L. equestris* oviposits on the ground-layer vegetation in June and July. Adults of the new generation commonly appear from late-July onwards ([24], L. Laukkanen, pers. obs.).

### Study populations

**Figure 1:**
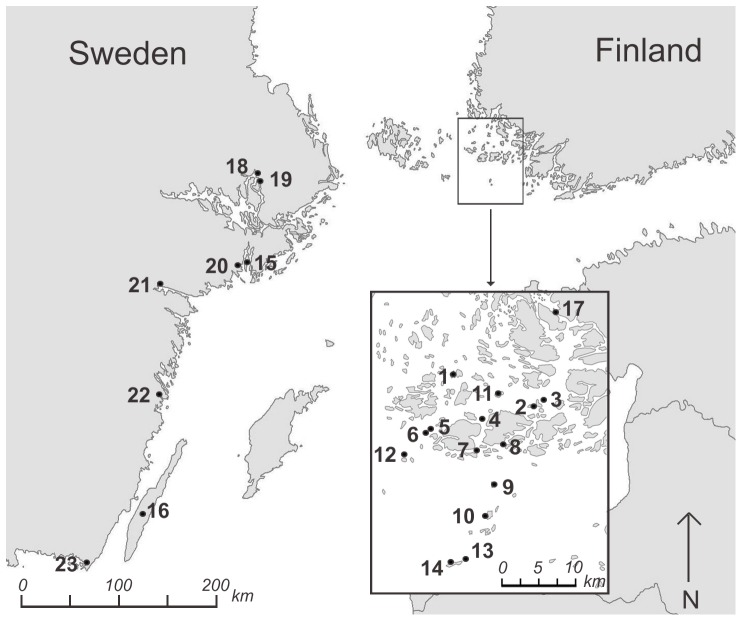
Location of 23 *Lygaeus equestris* study sites.

Samples for genetic analysis were collected from 23 *L. equestris* populations in June and July 2007. Fifteen of the populations are located in southwestern Finland and eight in eastern Sweden (Table 1, Fig. 1). One Finnish and six Swedish populations are located on the coastal mainland, and the rest of the populations are located on separate islands of the Baltic Sea. We collected on average 27.3±1.12 (mean ± se) individuals from each population (628 individuals in total). The samples were stored in 96.1% ethanol and kept at 4°C before DNA extraction.

**Table 1:** Characteristics of the 23 study sites.

Study site	Island size (ha)	Population size of	Population size of	Number of plant species
		*L. equestris*	*V. hirundinaria*	
Islands:				
1 Åvensor	546.1	637	1 100	51
2 Lammasluoto	13.0	1 053	5 000	40
3 Jäämäluoto	5.8	8	55	23
4 Ånskär	3.8	526	2 500	22
5 Limskär	3.5	100	2 000	43
6 Alskär	3.6	40	800	47
7 Killingholm	40.4	200	1 000	40
8 Petsor	85.5	28	80	27
9 Berghamn	62.5	40	120	36
10 Grisselharuna	3.4	48	475	31
11 Innamo	274.8	.	83	26
12 Birskär	28.0	.	31	22
13 Sanden	7.9	.	500	28
14 Jurmo	281.5	.	1 800	36
15 Mörkö	5 409.8	.	>6 000	62
16 Resmo alvar	13 8793.1	.	>12 000	36
Mainland sites:				
17 Naantali	.	.	800	51
18 Morga	.	.	30	32
19 Vassundaberget	.	.	175	45
20 Tullgarn	.	.	240	51
21 Getå	.	.	350	46
22 Verkebäck	.	.	200	48
23 Jämjö	.	.	135	61

*Vincetoxicum hirundinaria* is the primary food-plant species of *Lygaeus equestris*. The number of plant species is the number of all vascular plant species present in the study sites excluding species from family Poaceae and genus *Carex*.

All necessary permits were obtained for the described study. Forest administration of Finland (Metsähallitus) granted a licence to collect *L. equestris* from the area of Archipelago national park. Our studies did not involve endangered or protected species.

### DNA extraction and genetic analysis

DNA of *L. equestris* was extracted with NucleoSpin Tissue kit (Macherey-Nagel, Düren, Germany) according to the manufacturer's standard protocol. All laboratory work and genotyping were performed by the Center of Evolutionary Applications (University of Turku, Finland). The genotyping was based on amplified fragment length polymorphism (AFLP) profiles that were generated using a protocol modified from Vos *et al.* [29]. This method has been used in many former population genetic studies of insects (e.g. [30]–[32]). AFLP is based on selective PCR amplification of restriction fragments from a total digest of genomic DNA. The amplification is done by using adapter sequences as target sites for primer annealing. AFLP products are separated according to size using gel electrophoresis. The AFLP band patterns may be used for monitoring the degree of similarity or differentiation among populations, and as the band patterns map to specific loci, the individuals can be genotyped based on the alleles they carry. The visualisation of genetic differences among individuals and populations using AFLP as molecular marker tool does not require prior knowledge about nucleotide sequence [29], [33]. For detailed description of methods see Supporting Information S1.

Marker reproducibility and genotyping-error rate were estimated using replicates in different phases of the AFLP protocol (Supporting Information S1). It is recommended that the number of running replicates is 5–10% of total number of samples [33]–[35]. We analysed duplicate AFLP profiles of 38 samples, which is 6.1% of the total number of samples. The replicates were run as separate reactions starting from separate DNA extractions, so that the error rates were reflective of the entire genotyping process. After DNA extraction the AFLP protocol was performed on 96 well plates, and positive controls were used to monitor for differences among the plates. We had four control samples on every plate in randomised positions (the same four samples on each plate: two samples were of ligated DNA, and two samples were of DNA). Usually the error rates fall in a 2–5% range [33], [34], [36]. In this study, the error rates were 0.1–0.5%, and thus the results were highly reproducible. Loci with a varying fragment size and potentially overlapping loci were discarded. In total, we used 86 loci in the further analyses.

#### Detecting non-variable and non-independent loci

Allele frequencies were calculated with the square-root method [37] to delete loci showing less than 5% variation, i.e., the proportion of an allele either present or absent was less than 5%. In addition, all loci were checked for linkage equilibrium (independence of loci) to be able to remove loci expressing patterns similar to other loci [17], [34]. This was done according to the formula presented in Gaudeul *et al.* [38]. For the non-independent loci pairs (less than 5% difference among loci), the locus showing less variation was deleted from the final data set. In many cases the non-independent loci also showed lack of variation. In total, 27 loci and 21 individuals were removed from the data, and we used 59 polymorphic loci and 607 individuals in the final data set.

#### Estimation of genetic differentiation and within-population genetic variation

We estimated the levels of genetic differentiation and within-population genetic variation of *L. equestris* using the AFLP-SURV 1.0 program [39]. We assumed that each marker had only two alleles (a dominant marker allele coding for the presence of a band, and a recessive null allele coding for the absence of the band) and assumed Hardy-Weinberg genotypic proportions. The allele frequencies (p and q) at each marker locus were calculated with the Bayesian method with non-uniform prior distribution of allele frequencies [40]. The frequency of the recessive null allele (q) at each locus was computed using the number of individuals in the sample that lacked the AFLP band and the sample size. The distribution of allele frequencies for each population was then estimated based on the variation over loci, and the statistics of genetic differentiation and within-population genetic variation were computed according to Lynch and Milligan [41]. We used the percentage of polymorphic loci (PLP) at 5% level and expected heterozygosity over all loci (Nei's gene diversity, H_e_) as measures of within-population genetic variation for each of the 23 populations [16]. To estimate H_e_, we first calculated the expected heterozygosity per locus, h_j_, by subtracting the expected frequencies of homozygotes from 1 (h_j_ = 1 – p^2^ – q^2^, where p and q are the allele frequencies). This was repeated for all loci and the average was calculated to obtain expected heterozygosity over all loci, H_e_. We also estimated the average genetic variation within populations by calculating the average PLP and mean expected heterozygosity, H_S_, and standard errors for these values.

In addition to estimating the level of within-population genetic variation, we calculated average Wright's fixation index, F_ST_, to estimate genetic differentiation among populations in terms of allele frequencies [16]. To be able to evaluate genetic differentiation between the individual *L. equestris* populations, we also calculated pairwise genetic differentiation as pairwise F_ST_ for all population pairs.

### Factors affecting genetic differentiation among and variation within *L. equestris* populations

The size of *V. hirundinaria* populations varies in our study area. Thus, the number of mature *V. hirundinaria* individuals was counted in all those study sites, where population size was lower than 100 individuals (Table 1). In populations with more than 100 individuals population sizes were estimated independently by two or three researchers. In most cases, each island harboured a single population. More than one *V. hirundinaria* population occur on the two largest islands (Mörkö, and Öland where the Resmo alvar population is located). On these two islands, the *L. equestris* samples were collected only from one site, and *V. hirundinaria* population size was estimated based on observations from this site [42], [43].

To test whether genetic differentiation between populations or within-population genetic variation of *L. equestris* was related to the diversity of the plant community we estimated plant-species diversity in the communities where the *V. hirundinaria* and *L. equestris* populations occurred. We conducted the vegetation survey twice during the summer 2009 (the first survey in late May – early June, the second in late August – early September) in all study sites, and all the plant species present at least once were regarded as present. Poaceae and *Carex* were excluded from the vegetation survey because of limited resources and the fact that these groups are not among the most important alternative food sources of *L. equestris* [24]. We have never observed *L. equestris* feeding on these plants in our study area (L. Laukkanen, A. Muola and R. Leimu, pers. obs.). Poaceae was abundant in all populations, but *Carex* species were present only in some of the populations. First, we investigated whether the genetic differentiation between the *L. equestris* populations was related to the similarity of vegetation between the study sites. For this analysis, we calculated the Sørensen's species similarity coefficients for each population pair using presence/absence data on the plant species [44]. Secondly, we tested whether within-population genetic variation of *L. equestris* correlated positively with the number of vascular plant species (see statistical analysis).

In August – September 2007 we estimated the population size of *L. equestris* in the same area where the *V. hirundinaria* population size was estimated. The population size of *L. equestris* was estimated by calculating the average number of individuals observed on 14–20 randomly chosen *V. hirundinaria* plants in the ten Finnish populations and multiplying the average by the estimated number of *V. hirundinaria* individuals in a population. The *V. hirundinaria* individuals from which *L. equestris* was counted varied in size and were chosen haphazardly from the entire area where *V. hirundinaria* occurred. The *L. equestris* individuals were counted in August – September to insure that all individuals represented the same generation. We then tested whether large *L. equestris* populations had more within-population genetic variation compared to smaller populations (see statistical analysis).

To investigate whether the genetic differentiation between the *L. equestris* populations was related to the geographic distance between populations we determined the distances for all population pairs using the geographic coordinate data registered with GPS (Garmin eTrex). The distances between the populations vary from one to 598 kilometres. We also examined whether the island populations had less within-population genetic variation than mainland populations, as water may restrict migration between populations [7], [16]. To test whether within-population genetic variation was related to island size [45] we determined the area of the islands using electronic maps (http://www.paikkatietoikkuna.fi/web/fi/kartta and http://www.gis.lst.se/lanskartor).

### Statistical analysis

We tested the significance of the overall genetic differentiation among populations with permutation tests. The resampling statistics was based on 100 000 random permutations. F_ST_ values were computed after each permutation consisting of randomly permuting individuals among existing populations. The real observed F_ST_ was then tested against the distribution obtained by permutation. The permutation tests for genetic differentiation were done using AFLP-SURV 1.0 program [39].

We tested whether the genetic differentiation of *L. equestris* populations (pairwise F_ST_) was related to the similarity of vegetation between the sites. We first tested whether the geographically closely located populations were more similar in vegetation by comparing the geographic distance with the Sørensen's similarity coefficients by simple Mantel test between the two matrices. Because geographically close populations were more similar in vegetation (r = −0.304, n = 253, *p* = 0.008), we tested whether the genetic differentiation of *L. equestris* was associated with the similarity of vegetation by conducting partial Mantel test correcting for the geographic distances between the populations. We also tested whether the genetic differentiation between the *L. equestris* populations correlated with the geographic distance between populations with simple Mantel test between the two matrices. The tests of significance were based on 100 000 random permutations [46] and were conducted using the zt statistical package [47].

We tested whether the within-population genetic variation of *L. equestris* was associated with geographic and ecological characteristics of the study sites. We used the percentage of polymorphic loci (PLP) and expected heterozygosity (H_e_) as estimates of within-population genetic variation [16]. We conducted separate mixed model ANOVAs with PLP or H_e_ as the dependent variables. The location of population on an island or on mainland was included as a fixed factor and *V. hirundinaria* population size and the number of vascular plant species were included as continuous factors in the analyses of the 23 *L. equestris* populations from Finland and Sweden. Because we had some additional data collected exclusively from the ten Finnish island populations, we conducted separate analyses using the data from these populations (populations 1–10; Table 1). We conducted separate general linear models for PLP and H_e_. The number of plant species, island size (area in ha), *L. equestris* population size, and the interactions between these variables were included as continuous variables in the analyses. The continuous explanatory variables were not correlated (data not shown) except for *V. hirundinaria* population size and *L. equestris* population size (Spearman correlation coefficients; r = 0.918, n = 10, *p*<0.001). This is due to the fact that *V. hirundinaria* population size was used to count *L. equestris* population size. The population sizes of *V. hirundinaria* and *L. equestris* were not included in same models. Because the assumptions of general linear model were not fulfilled for PLP in the analysis with the 10 *L. equestris* populations from Finland, these data were analysed using ranked values. We used the AIC values to select the best-fitting models in all analyses [48]. The final models consisted of factors with positive or neutral effects on model stability according to the AIC values. The complete models without any model simplification are presented in the Supporting Information S2. The analyses were conducted using the MIXED procedure of the SAS Statistical Package (version SAS 9.2) (SAS Institute Inc. 2002–2007).

## Results

### Population genetic differentiation

The pairwise F_ST_ values ranged from 0 to 0.100. This level of among-population differentiation can be considered low or moderate [19], [49]. In total, the F_ST_ values of 45 population pairs (17.7% of all pairs) indicated moderate differentiation (F_ST_ between 0.05 and 0.15). The average F_ST_ indicating overall genetic differentiation among all populations was 0.034±0.001 (mean ± se, 253 pairwise F_ST_ values included). This value suggests rather low genetic differentiation among the *L. equestris* populations. However, the permutation test indicated that populations were genetically more differentiated than random assemblages of individuals (*p*<0.05).

**Figure 2:**
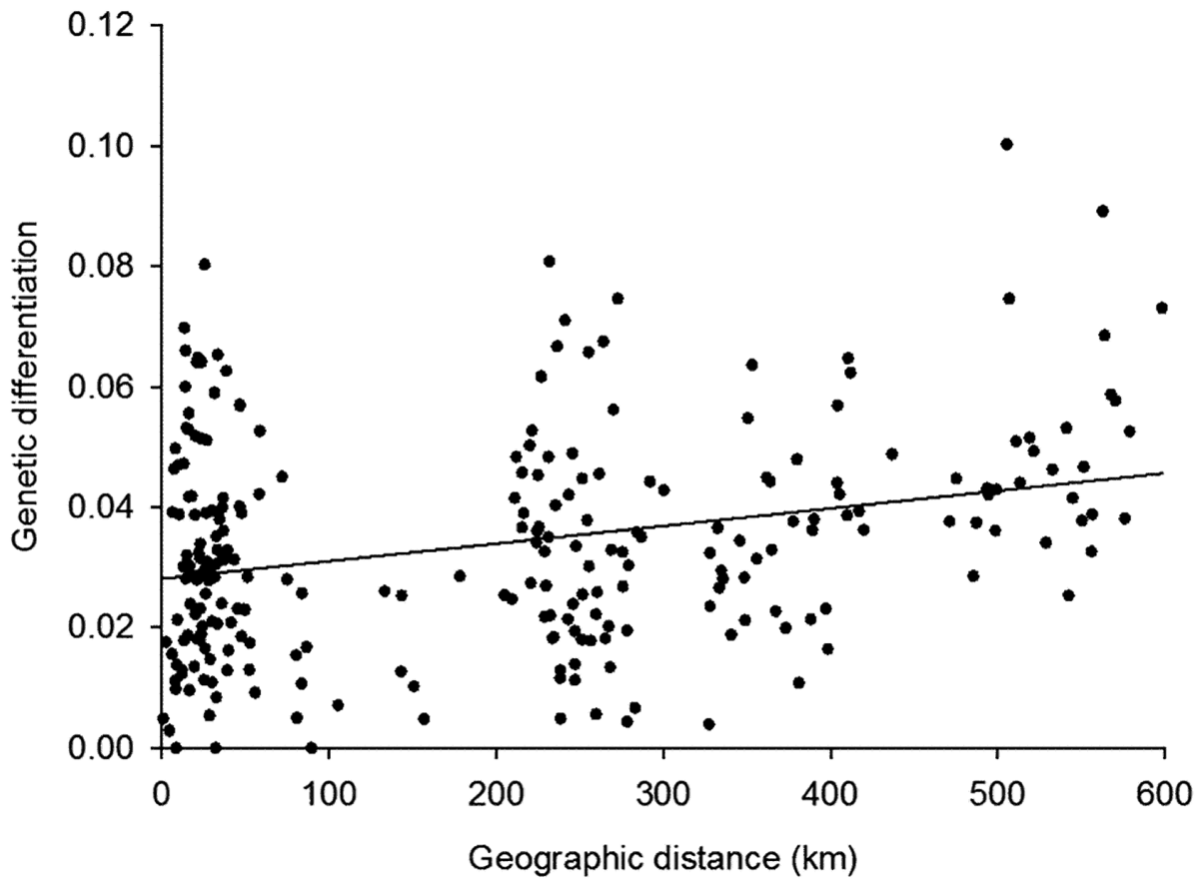
Correlation of genetic differentiation and geographic distance among *Lygaeus equestris* populations. Genetic differentiation was estimated as pairwise F_ST_.

Genetic differentiation between the *L. equestris* populations was not related to the similarity of vegetation between the study sites (F_ST_ vs. Sørensen's similarity coefficients, partial Mantel test: r = 0.122, n = 253, *p* = 0.361). Genetic differentiation of the *L. equestris* populations increased with increasing geographic distance between the populations (F_ST_ vs. geographic distance, Mantel test: r = 0.288, n = 253, *p* = 0.026; Fig. 2).

### Within-population genetic variation

We found significant level of genetic variation within the *L. equestris* populations. The percentage of polymorphic loci (PLP) ranged from 74.6% to 94.9% in the individual populations (Table 2). The average PLP of all 23 populations was 86.0±1.32% (mean ± se). The level of expected heterozygosity within the populations (Nei's gene diversity, H_e_) ranged from 0.265 to 0.316 (Table 2). The mean expected heterozygosity (mean Nei's gene diversity, H_S_) was 0.294±0.003 (mean ± se).

**Table 2:** Population genetic characteristics of *Lygaeus equestris*.

Population	Number of samples	PLP	H_e_	S.E. (H_e_)
islands:				
1	29	86.4	0.291	0.022
2	29	83.1	0.297	0.021
3	28	86.4	0.288	0.019
4	28	74.6	0.269	0.022
5	29	86.4	0.309	0.021
6	29	78.0	0.291	0.022
7	29	91.5	0.311	0.019
8	29	76.3	0.265	0.022
9	15	94.9	0.306	0.019
10	29	88.1	0.288	0.018
11	25	89.8	0.292	0.019
12	21	88.1	0.296	0.020
13	29	88.1	0.298	0.020
14	28	94.9	0.316	0.018
15	29	89.8	0.304	0.019
16	29	91.5	0.291	0.019
mainland:				
17	8	76.3	0.278	0.023
18	29	78.0	0.276	0.022
19	28	78.0	0.272	0.021
20	29	93.2	0.311	0.019
21	29	93.2	0.298	0.018
22	27	86.4	0.305	0.020
23	22	84.7	0.307	0.021

PLP is the percentage of polymorphic loci at the 5% level, H_e_ is expected heterozygosity (Nei's gene diversity) under Hardy-Weinberg genotypic proportions, and S.E. (H_e_) is the standard error of expected heterozygosity.

### Effects of ecological and geographic factors on within-population genetic variation

When all 23 populations from Finland and Sweden were included in the analysis, neither the population size of *V. hirundinaria* nor the number of vascular plant species affected the percentage of polymorphic loci, PLP (F_1,17_ = 0.46, *p* = 0.507 and F_1,17_ = 1.07, *p* = 0.315, respectively). PLP did not vary significantly between island and mainland populations (F_1,17_ = 0.51, *p* = 0.486). Furthermore, the effect of the population size of *V. hirundinaria* on PLP of *L. equestris* did not differ between island and mainland populations (*V. hirundinaria* population size by island/mainland, F_1,17_ = 0.50, *p* = 0.489). Moreover, the effect of the number of plant species on PLP did not differ between island and mainland populations (island/mainland by number of plant species, F_1,17_ = 0.38, *p* = 0.546).

**Figure 3:**
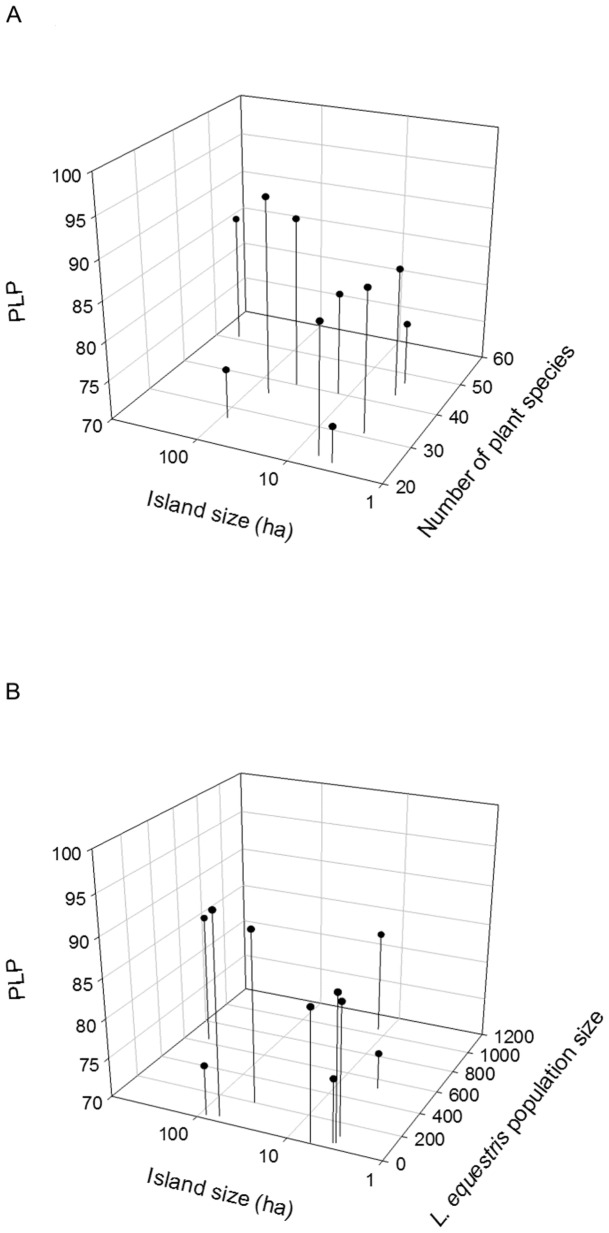
Interactive effects of plant diversity, island size, and population size on the percentage of polymorphic loci. 3a) Interactive effect of plant-species diversity (number of vascular plant species) and island size on the percentage of polymorphic loci (PLP) of *Lygaeus equestris*. 3b) Interactive effect of island size and population size on PLP of *L. equestris*.

When the percentage of polymorphic loci, PLP, was tested separately using the ten island populations from Finland, the two-way interaction between the number of plant species and the island size was significant indicating that the effect of plant-species diversity on PLP of *L. equestris* was related to island size (Table 3, Fig. 3a). On large islands, PLP seemed to be higher when plant-species diversity was high. On small islands plant-species diversity was not related to within-population genetic variation of *L. equestris* in terms of PLP. Furthermore, the two-way interaction between island size and the population size of *L. equestris* was significant (Table 3, Fig. 3b). This suggests that the effect of the population size of *L. equestris* on PLP was related to island size. When a small *L. equestris* population occurred on a small island, the percentage of polymorphic loci was on the average low, whereas small populations on larger islands seemed to maintain a higher level of within-population genetic variation (Fig. 3b). In addition, the main effect of island size on PLP of *L. equestris* was significant: the larger the island, the higher was the percentage of polymorphic loci (Table 3).

**Table 3:** Results of a general linear model on the effects of number of vascular plant species, island size, and population size of *Lygaeus equestris* on the percentage of polymorphic loci of *L. equestris*.

Source of variation	MS	F	*p*
Number of plant species	10.54	3.16	0.173
Island size	45.94	13.79	0.034
*L. equestris* population size	20.93	6.28	0.087
Number of plant species * Island size	47.27	14.19	0.033
Number of plant species * *L. equestris* pop.	24.65	7.40	0.073
Island size * *L. equestris* pop. size	44.71	13.42	0.035

The analysis included data from the 10 island populations from the Finnish Archipelago (Table 1). The degrees of freedom for all factors are 1 and 3.

**Figure 4:**
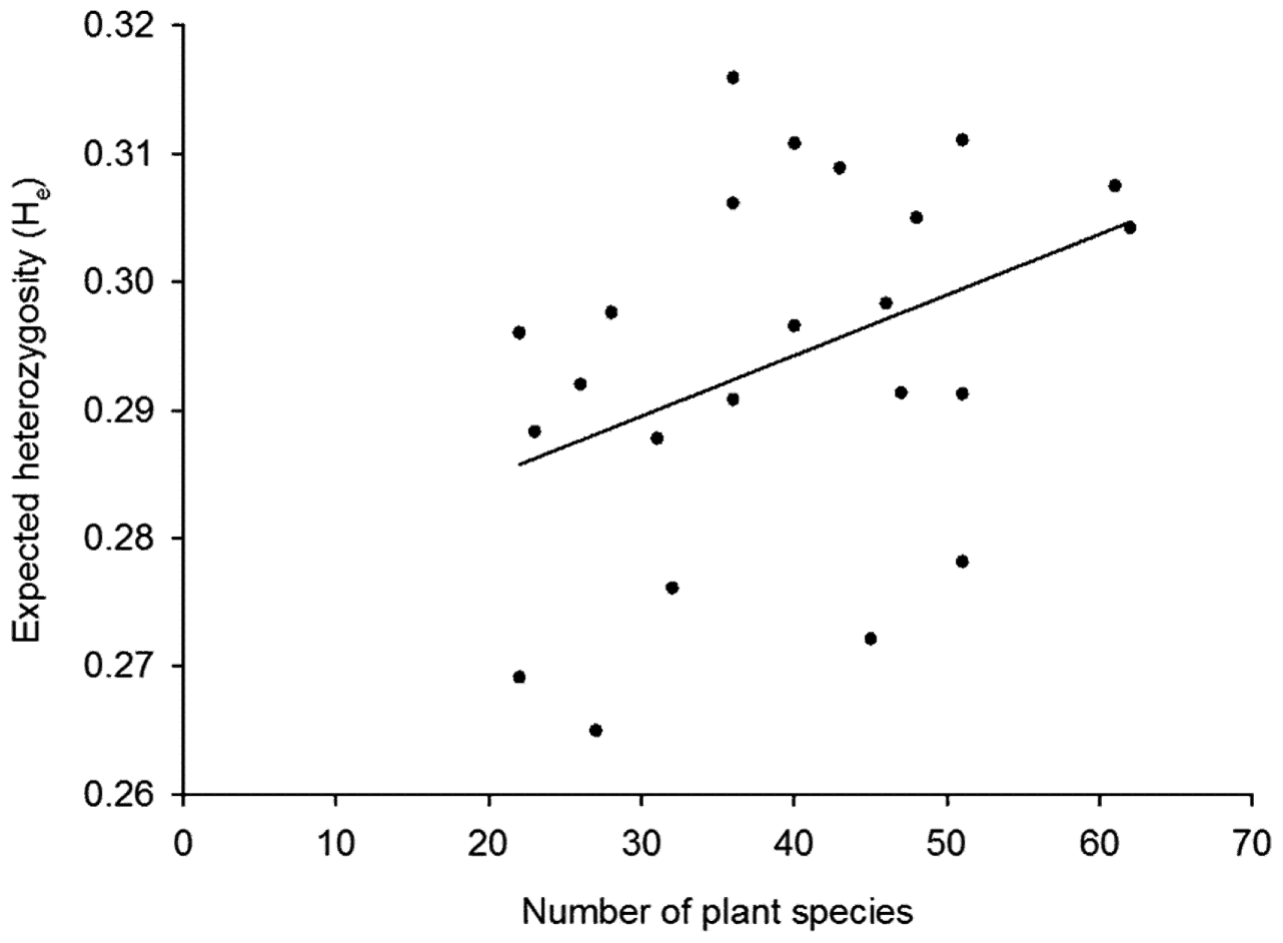
Effect of plant-species diversity on expected heterozygosity (H_e_) of *Lygaeus equestris*. Plant-species diversity was estimated as the number of vascular plant species.

When all 23 populations from Finland and Sweden were included in the analysis, the population size of *V. hirundinaria* did not influence expected heterozygosity (H_e_) of *L. equestris* (F_1,19_ = 0.38, *p* = 0.547). In contrast, the effect of the number of vascular plant species on H_e_ was significant (F_1,19_ = 6.24, *p* = 0.022; Fig. 4): when the diversity of the plant community increased also the level of genetic variation within *L. equestris* population in terms of H_e_ increased (Fig. 4). H_e_ did not vary significantly between island and mainland populations (F_1,19_ = 2.44, *p* = 0.135).

When the ten island populations from Finland were analysed separately, the effect of the number of vascular plant species on expected heterozygosity, H_e_, was significant (F_1,6_ = 6.17, *p* = 0.048). As in the model with all 23 populations, the more plant species in the plant community, the higher was the level of genetic variation within *L. equestris* population in terms of H_e_. Neither island size, nor the population size of *L. equestris* affected H_e_ (F_1,6_ = 1.48, *p* = 0.270 and F_1,6_ = 0.10, *p* = 0.762, respectively).

## Discussion

### The effect of vegetation on within-population genetic variation of an oligophagous herbivore

In line with our predictions, our results demonstrate that in terms of expected heterozygosity (Nei's gene diversity, H_e_) genetic variation within the populations increased with the diversity of the plant community. However, the effect of plant-species diversity on the percentage of polymorphic loci depended on island size. The diverse set of alternative food-plant species together with other resources (i.e. more diverse habitats for oviposition and hibernation) occurring on large islands may enable the existence of highly viable and stable *L. equestris* populations with high level of within-population genetic variation. On large islands with high plant-species diversity and high within-population genetic variation *L. equestris* may be able to feed on several alternative species simultaneously. These populations may then survive through periods with no *V. hirundinaria* seeds available. Agrawal *et al*. [50] and Züst *et al.* [51] recently showed that selection by the herbivore communities modify the genetic structure of their food-plant populations. Our results suggest that the plant community might also affect the population genetics of the herbivore species.

In addition to the availability of a diverse set of alternative food-plant species, the availability of the primary food, *V. hirundinaria* seeds, is assumed to be essential for survival and reproduction of *L. equestris*. In general, seed production is assumed to be higher in large plant populations compared to smaller ones, and large plant populations may support large herbivore populations that have higher level of within-population genetic variation [11], [12]. The fact that the level of genetic variation within the *L. equestris* populations was not influenced by the population size of *V. hirundinaria* could be due to the seed-production strategy of *V. hirundinaria*. The seed production of *V. hirundinaria* varies considerably among populations and among years mainly due to variation in sun exposure ([25], [52], L. Laukkanen, unpubl. data). *Lygaeus equestris* populations in Sweden have been observed to decrease when *V. hirundinaria* seeds are abundant and increase when seeds are scarce [53], [54]. The reason for this paradox is that weather conditions affect both *L. equestris* and seed resources directly, and these effects are negatively correlated: *L. equestris* thrives in dry and hot summers, whereas *V. hirundinaria* suffers from drought and high temperature [28]. The amount of seeds produced in the previous year is important if the summer is warm and dry [53], [54]. Thus, in years of high seed production even a small number of *V. hirundinaria* individuals may be able to produce enough seeds to maintain an entire *L. equestris* population. In these years, the population size of *L. equestris* may be limited by cold weather [26], [28]. On the other hand, when the seed production of *V. hirundinaria* is low in general, all individual *V. hirundinaria* plants within a population are likely to produce a low number of seeds or no seeds at all regardless of population size, and the *L. equestris* population suffers from a shortage of the primary food possibly leading to a population bottleneck and consequently to reduced within-population genetic variation. In addition to weather conditions, variation in the abundance of the other specialist seed predator of *V. hirundinaria*, the tephritid fly *Euphranta connexa* (L. Laukkanen, unpubl. data), affects the abundance of *V. hirundinaria* seeds. Consequently, the potential competitive effect of *E. connexa* on *L. equestris* is likely to vary among populations ([11], [25], L. Laukkanen, unpubl. data). Another possible explanation for the fact that the level of genetic variation within the *L. equestris* populations was not influenced by the population size of *V. hirundinaria* is the impact of contemporary migration (see below).

We used plant-species diversity and the abundance of the primary food plant as features of the plant community to explain genetic variation of an oligophagous herbivore. In nature, in addition to the number of the plant species, their spatial distribution, densities, and relative abundances contribute to the ability of herbivores to find and use the different food plants [55]. Although being a robust measure, plant-species diversity is probably a suitable indicator of the food plants available in a given habitat for oligophagous and generalist herbivores, whereas other, more specific characteristics of the plant community are important for specialist herbivores. In general, herbivores search their food plants using visual and/or olfactory cues (i.e. plant volatiles) [56]. We do not currently know how *L. equestris* finds its food plants. However, another Lygaeid species, *Oncopeltus fasciatus*, finds its food plant (*Asclepias* spp., Apocynaceae) mainly by olfactory cues and prefers dense patches of food plants [57], [58]. Therefore, it seems likely that olfactory cues are also used for locating suitable food plants by our study species, *L. equestris*.

### The effect of geographic factors and population size on within-population genetic variation

Compared to other sexually reproducing insects, the *L. equestris* populations studied here have a high level of within-population genetic variation (e.g. [30], [31]). Migration seems to be high enough to sustain the high levels of within-population genetic variation even in the island populations. *Lygaeus equestris* is well able to fly distances of few kilometers [26], and we have occasionally encountered individuals even on islands where the primary food plant *V. hirundinaria* is not present (A. Muola, pers. obs.).

In general, small populations often have less within-population genetic variation compared to large populations due to loss of alleles caused by inbreeding and genetic drift [16], [18], [19], [59]. We found that small *L. equestris* populations on small islands had low percentage of polymorphic loci on average, but small populations on larger islands harboured a higher level of within-population genetic variation. In our study area, *L. equestris* populations on large islands may be less susceptible to population bottlenecks and extinction-recolonisation processes that both can alter allele frequencies in populations and reduce within-population genetic variation [19]. Firstly, populations in large islands may be less prone to extinctions and recolonisations compared to smaller islands due to more stable microclimates or more diverse habitats for feeding, oviposition, and hibernation. Secondly, in our study area, island size correlates positively with island age (L. Laukkanen, unpubl. data) suggesting that *L. equestris* populations on large islands are likely to be older than those on smaller islands. The small but older *L. equestris* populations on larger islands are likely to have had a longer history of inbreeding suggesting that deleterious recessive alleles have been purged in these populations. However, if the small and young populations on small islands still harbour deleterious alleles that are expressed due to inbreeding, these populations may be more prone to extinction [19], [59]. Thus, extinction-recolonisation processes may lead to reduced level of within-population genetic variation especially in the small and young populations.

### Population genetic differentiation

Geographical isolation is an important determinant of genetic differentiation (e.g. [60]). Consistent with this, we found that genetic differentiation of *L. equestris* increased with increased geographic distance among the populations (Fig. 2). The reason for the four horizontal clusters evident in Fig. 2 is that there are no population pairs with certain distances in our study area because of the large open sea area (see Fig. 1). However, the data points are scattered evenly relative to the y-axis (no gaps in distribution of genetic differentiation) providing evidence for isolation by distance (Fig. 2) (see [61]). Peterson and Denno [8] found that isolation by distance was strongest in moderately mobile insect species. This is logical, because highly mobile species may have extensive gene flow over both small and large distances, and in sedentary species gene flow is weak across the entire spatial scale [8]. *Lygaeus equestris* may infrequently fly long distances, but usually the geographic scale of migration is few kilometres maximum [26], [62], [63]. Thus, *L. equestris* could be classified as a “moderately mobile” species that is likely to show isolation by distance, which was also observed here. However, the only low or moderate level of differentiation observed among the *L. equestris* populations may be explained not only by high migration, but also by the relatively short history of the *L. equestris* populations in the area of the Baltic Sea. In general, populations that have persisted over long time are expected to be more differentiated than younger populations [19]. The effects of genetic drift, accumulation of mutations, and/or differential selection may have acted for such a short period of time that their effects on genetic differentiation among the *L. equestris* populations are likely to have been minor.

Wang *et al*. [60] recently stated that geographical isolation explains genetic differentiation more than ecological isolation. Our results support this notion since we found that in contrast to the geographic distance, the similarity of vegetation was not related to the population differentiation of *L. equestris*. This, together with the result that the number of plant species present in a habitat was related to the level of within-population genetic variation (H_e_), seems to suggest that the number of potential alternative food-plant species present in a habitat might be more important for survival of *L. equestris* than the identity of species *per se*. Indeed, most plant species have been shown to be quite poor quality food for *L. equestris*, and lead to reduced growth and lower reproduction [5], [64]. Population differentiation of *L. equestris* has previously been studied in Sweden using allozyme electrophoresis [65]. In line with our results, Sillén-Tullberg [65] found genetic differentiation of *L. equestris* between her two study areas (225 km distance), but not among the seven populations within one area (average distance between the populations was 3.5 km; F_ST_ = 0.010). In our study, the pairwise F_ST_ values of *L. equestris* population pairs with less than 10 km distance indicated low level of differentiation (n = 13, all pairwise F_ST_<0.05).

Our findings provide valuable information about the role of ecological and geographic factors as modifiers of the population genetic structure of an oligophagous seed-eating herbivore. Several studies have shown that herbivore-species diversity increases with plant-species diversity and plant genetic diversity (e.g. [66], [67]). To our knowledge, our study is the first to suggest that plant-species diversity is related to the population genetics of an herbivore species: the more diverse was the plant community, the higher was the level of within-population genetic variation of the seed predator. Whether the higher genetic variation of the seed predator is related to the damage the predators induce on their food plants would be worth further studies. The diversity of food organisms is likely to affect predator genetic variation in other plant-herbivore and predator-prey systems as well. Therefore we believe that our results have important, and previously not extensively studied implications for studies of trophic interactions, habitat fragmentation, and landscape genetics.

## Supporting Information

Supporting Information S1 **Description of DNA extraction and genetic analysis.**
(DOC)

Supporting information S2 **Results from mixed model ANOVAs and general linear models. Complete models without model simplification.**
(DOCX)
