# Supplementary material for: Plant-Species Diversity Correlates with Genetic Variation of an Oligophagous Seed Predator
Source: PLoS One. 2014 Apr 11;9(4):e94105. doi: 10.1371/journal.pone.0094105 (PMC3984091; doi:10.1371/journal.pone.0094105)
Supplement: Supporting Information S1 — Description of DNA extraction and genetic analysis. (DOC) [file pone.0094105.s001.doc]

LIISA LAUKKANEN, PIA MUTIKAINEN, ANNE MUOLA and ROOSA LEIMU

Plant-species diversity correlates with genetic variation of an oligophagous seed predator

**Supporting Information S1**

**Description of DNA extraction and genetic analysis**

DNA was extracted with NucleoSpin Tissue kit (Macherey-Nagel, Düren, Germany) according to the manufacturer’s standard protocol. All subsequent laboratory work and genotyping were performed by the Center of Evolutionary Applications (University of Turku, Finland). The genotyping was based on amplified fragment length polymorphism (AFLP) profiles that were generated using a protocol modified from Vos et al. (1995).

1. Restriction of the DNA. The DNA was digested with two restriction enzymes, Mse-I and EcoR-I (New England Biolabs). Mse-I is a frequent cutter with a 4 base recognition site, and EcoR-I is a rare cutter with a 6 base recognition site. The reaction consisted of restriction at 37 °C for 3 h and heat inactivation at 65 °C for 20 min using 100-700 µl of genomic DNA in a total reaction volume of 20 µl.

2. Ligation of adapters. Adapters that place a known sequence at the ends of the restriction fragments were then added together with T4 DNA ligase (Fermentas) to give a total reaction volume of 40 µl and incubated at 17 °C for 14 h. The reaction was then diluted 1:5 in distilled MQ-water.

3. Preselective PCR. Preselective primers are complementary to the adapter sequences with the addition of one nucleotide at the 3’ end to amplify only a subset of all DNA fragments. Two primer pairs with one selective nucleotide in each were used (Presel 1: Eco-A/Mse-C and Presel 2: Eco-C/Mse-C). The PCR reactions were performed in 20 µl (Presel 1 on a Ab 2720 thermal cycler, Applied Biosystems, Foster City, CA and Presel 2 on a PTC-100 thermal cycler, MJ Research, Watertown, MA) including 4 µl of digested and diluted gDNA, 0.5 units of Taq DNA polymerase (BioTaq, Bioline, London, UK), 0.4 and 0.8 µM of Eco- and Mse-primers respectively, 1 X PCR buffer (10 mM Tris-HCl, 50 mM KCl, pH 8.3), 0.2 mM of each dNTP and 1.5 mM MgCl_2_. PCR profile was as follows: 72 °C for 1 min followed by 20 cycles of 94 °C for 30 s, annealing at 56 °C for 1 min and extension at 72 °C for 60 s. Finally the reaction was diluted 1:19 in distilled MQ-water.

4. Selective PCR. This step used primers that match the known adaptor sequence, plus three selective nucleotides on the 3’ end of forward and reverse primers. Eco-primers were fluorescently labelled (Sel 1: FAM-Eco-ACA/Mse-CTG, Sel 2: NED-Eco-CTC/Mse-CGT). The PCR reactions were performed in 20 µl (Sel 1 on Ab 2720 and Sel 2 on PTC-100 thermal cycler) including 4 µl of diluted preselective PCR product, 0.5 units of Taq DNA polymerase, 0.4 and 0.6 µM of Eco- and Mse-primers respectively, 1 X PCR buffer, 0.2 mM of each dNTP and 1.5 mM MgCl_2_. A touchdown PCR with 35 cycles was used. The PCR protocol started with 12 cycles of 95 °C for 30 s, annealing at 60 °C for 30 s and extension at 72 °C for 1 min. After each cycle the annealing temperature was decreased by 0.7 °C. The next 23 cycles were performed at annealing temperature 56°C and the last cycle was followed by a final extension at 72 °C for 10 min.

For the fragment analysis by capillary electrophoresis the FAM- and NED-labelled PCR products were pooled and diluted (1:50), 2 µl of this was combined with 10 µl formamide/GS600LIZ-mix and run on a Abi3130xl Genetic Analyzer (Applied Biosystems). Multilocus profiles were visualised using GeneMapper 4.0 (Applied Biosystems).The scoring system was automated but each genotype was also inspected and the scoring confirmed visually. The scoring parameters were optimized by determining a locus specific peak amplitude thresholds for absence/presence of a fragment (thresholds varied between 50-300 intensity units). In order to maximise fragment homology within each loci only fragments ranging from 100 to 400 bp were used and of these only loci that had clear and distinct fragments (max bin width 1 bp). Loci with a varying fragment size or potentially overlapping loci were discarded. To minimise peak height variation between different analysis times (plates) we used the normalization method provided in GeneMapper (scope within project, method sum of signal).

Marker reproducibility and genotyping error rate were estimated using replicates in different phases of the AFLP protocol. After DNA extraction the AFLP protocol was performed on 96 well plates and positive controls were used to monitor for differences between plates. We had 4 control samples on every plate in randomized positions (same 4 samples on each plate: 2 samples were of ligated DNA and 2 samples were of DNA). In addition, we did duplicate AFLP profiles of 38 samples (separate reactions starting from separate DNA extractions).

**Table 1.** Genotyping error rates.

|  | **No. of samples replicated** | **No. of replications** | **No. of loci** | **Total allele count** | **No. of differences** | **Error rate** | **Genotyping score** |
| --- | --- | --- | --- | --- | --- | --- | --- |
| Among plates (PCR, fragment analysis) | 2 | 7-8 | 86 | 1286 | 3 | 0.002 | 0.998 |
| Among plates (AFLP procedure) | 2 | 7-9 | 86 | 1458 | 7 | 0.005 | 0.995 |
| Within plate (DNA quality) | 38 | 2 | 86 | 6491 | 6 | 0.001 | 0.999 |

Vos P, Hogers R, Bleeker M, Reijans M, van de Lee T, et al. (1995) AFLP: a new technique for DNA ﬁngerprinting. Nucleic Acids Res 23: 4407–4414.
