# Supplementary material for: Plant-Species Diversity Correlates with Genetic Variation of an Oligophagous Seed Predator
Source: PLoS One. 2014 Apr 11;9(4):e94105. doi: 10.1371/journal.pone.0094105 (PMC3984091; doi:10.1371/journal.pone.0094105)
Supplement: Supporting information S2 — Results from mixed model ANOVAs and general linear models. Complete models without model simplification. (DOCX) [file pone.0094105.s002.docx]

LIISA LAUKKANEN, PIA MUTIKAINEN, ANNE MUOLA and ROOSA LEIMU

Plant-species diversity correlates with genetic variation of an oligophagous seed predator

**Supporting information S2. Results from mixed model ANOVAs and general linear models. Complete models without model simplification.**

1. Results of an ANOVA model on the effects of population size of primary food plant *Vincetoxicum hirundinaria*, location of population on an island or on mainland, and the number of vascular plant species on the percentage of polymorphic loci (PLP) of *Lygaeus equestris.* 23 *L. equestris* populations were included in the analysis. The degrees of freedom for all factors are 1 and 15.

| **Source of variation** | **MS** | **F** | ***p*** |
| --- | --- | --- | --- |
| *V. hirundinaria* population size | 80.61 | 1.74 | 0.207 |
| Island / mainland | 102.45 | 2.22 | 0.157 |
| Number of plant species | 96.56 | 2.09 | 0.169 |
| *V. hirundinaria* population size * Island / mainland | 81.50 | 1.76 | 0.204 |
| *V. hirundinaria* pop. size * Number of plant species | 85.72 | 1.85 | 0.194 |
| Island / mainland * Number of plant species | 88.28 | 1.91 | 0.187 |
| *V. hirundinaria* pop. size * Island / mainland * No. of plant species | 87.27 | 1.89 | 0.190 |

1. Results of a general linear model on the effects of the number of vascular plant species, island size, and population size on the percentage of polymorphic loci (PLP) of *Lygaeus equestris.* 10 *L. equestris* populations were included in the analysis. The degrees of freedom for all factors are 1 and 2.

| **Source of variation** | **MS** | **F** | ***p*** |
| --- | --- | --- | --- |
| Number of plant species | 9.94 | 2.33 | 0.266 |
| Island size | 45.58 | 10.70 | 0.082 |
| *L. equestris* population size | 22.40 | 5.26 | 0.149 |
| Number of plant species * Island size | 39.48 | 9.27 | 0.093 |
| Number of plant species * *L. equestris* population size | 17.90 | 4.20 | 0.177 |
| Island size * *L. equestris* population size | 0.55 | 0.13 | 0.754 |
| Number of plant species * Island size * *L. equestris* pop. size | 1.47 | 0.35 | 0.616 |

1. Results of an ANOVA model on the effects of population size of primary food plant *Vincetoxicum hirundinaria*, location of population on an island or on mainland, and the number of vascular plant species on heterozygosity of *Lygaeus equestris.* 23 *L. equestris* populations were included in the analysis. The degrees of freedom for all factors are 1 and 15.

| **Source of variation** | **MS** | **F** | ***p*** |
| --- | --- | --- | --- |
| *V. hirundinaria* population size | 0.00002945 | 0.15 | 0.706 |
| Island / mainland | 0.00023606 | 1.19 | 0.293 |
| Number of plant species | 0.00091230 | 4.58 | 0.049 |
| *V. hirundinaria* population size * Island / mainland | 0.00002947 | 0.15 | 0.706 |
| *V. hirundinaria* pop. size * Number of plant species | 0.00004054 | 0.20 | 0.658 |
| Island / mainland * Number of plant species | 0.00017979 | 0.90 | 0.357 |
| *V. hirundinaria* pop. size * Island / mainland * No. of plant species | 0.00003998 | 0.20 | 0.660 |

1. Results of a general linear model on the effects of the number of vascular plant species, island size, and population size on heterozygosity of *Lygaeus equestris.* 10 *L. equestris* populations were included in the analysis. The degrees of freedom for all factors are 1 and 2.

| **Source of variation** | **MS** | **F** | ***p*** |
| --- | --- | --- | --- |
| Number of plant species | 0.00000574 | 0.06 | 0.833 |
| Island size | 0.00078260 | 7.82 | 0.108 |
| *L. equestris* population size | 0.00027072 | 2.70 | 0.242 |
| Number of plant species * Island size | 0.00062192 | 6.21 | 0.130 |
| Number of plant species * *L. equestris* population size | 0.00024295 | 2.42 | 0.260 |
| Island size * *L. equestris* population size | 0.00000783 | 0.08 | 0.806 |
| Number of plant species * Island size * *L. equestris* pop. size | 0.00002125 | 0.21 | 0.690 |
